# Systematic review of sedentary behaviour and health indicators in school-aged children and youth

**DOI:** 10.1186/1479-5868-8-98

**Published:** 2011-09-21

**Authors:** Mark S Tremblay, Allana G LeBlanc, Michelle E Kho, Travis J Saunders, Richard Larouche, Rachel C Colley, Gary Goldfield, Sarah Connor Gorber

**Affiliations:** 1 Healthy Active Living and Obesity Research, Children's Hospital of Eastern Ontario Research Institute. 401 Smyth Road, Ottawa, Ontario, K1H 8L1, Canada; 2 Department of Physical Medicine and Rehabilitation, Johns Hopkins University. 600 North Wolfe Street, Baltimore, Maryland, 21202, USA; 3 Office of the Task Force on Preventive Health Care, Public Health Agency of Canada. 785 Carling Avenue, Ottawa, Ontario, K1A 0K9, Canada

**Keywords:** Inactivity, sitting, TV, body composition, fitness, metabolic syndrome, cardiovascular disease, self-esteem, pro-social behaviour, academic achievement

## Abstract

Accumulating evidence suggests that, independent of physical activity levels, sedentary behaviours are associated with increased risk of cardio-metabolic disease, all-cause mortality, and a variety of physiological and psychological problems. Therefore, the purpose of this systematic review is to determine the relationship between sedentary behaviour and health indicators in school-aged children and youth aged 5-17 years. Online databases (MEDLINE, EMBASE and PsycINFO), personal libraries and government documents were searched for relevant studies examining time spent engaging in sedentary behaviours and six specific health indicators (body composition, fitness, metabolic syndrome and cardiovascular disease, self-esteem, pro-social behaviour and academic achievement). 232 studies including 983,840 participants met inclusion criteria and were included in the review. Television (TV) watching was the most common measure of sedentary behaviour and body composition was the most common outcome measure. Qualitative analysis of all studies revealed a dose-response relation between increased sedentary behaviour and unfavourable health outcomes. Watching TV for more than 2 hours per day was associated with unfavourable body composition, decreased fitness, lowered scores for self-esteem and pro-social behaviour and decreased academic achievement. Meta-analysis was completed for randomized controlled studies that aimed to reduce sedentary time and reported change in body mass index (BMI) as their primary outcome. In this regard, a meta-analysis revealed an overall significant effect of -0.81 (95% CI of -1.44 to -0.17, p = 0.01) indicating an overall decrease in mean BMI associated with the interventions. There is a large body of evidence from all study designs which suggests that decreasing any type of sedentary time is associated with lower health risk in youth aged 5-17 years. In particular, the evidence suggests that daily TV viewing in excess of 2 hours is associated with reduced physical and psychosocial health, and that lowering sedentary time leads to reductions in BMI.

## Introduction

Engaging in regular physical activity is widely accepted as an effective preventative measure for a variety of health risk factors across all age, gender, ethnic and socioeconomic subgroups [1-6]. However, across all age groups, levels of physical activity remain low [7-12] and obesity rates continue to rise [10,11,13,14]; collectively threatening the persistent increase in life expectancy enjoyed over the past century and efforts to counteract the inactivity and obesity crisis [15].

This inactivity crisis is especially important in the pediatric population as recent data from the Canadian Health Measures Survey [8] suggest that only 7% of children and youth aged 6-19 years participate in at least 60 minutes of moderate- to vigorous-intensity physical activity per day, thus meeting the current physical activity guidelines from Canada [16], the U.S. [6], the U.K [17], Australia [18] and the World Health Organization (WHO) [5]. However, even for those children and youth who meet current guidelines, there remains 23 hours per day for school, sleep, work, and discretionary time. Several sources report that children and youth spend the majority of their discretionary time engaging in sedentary pursuits (e.g. watching television (TV) or playing video games) [8,19-28]. Canadian children and youth are spending an average of 8.6 hours per day, or 62% of their waking hours being sedentary [8]. Similar trends are being reported in the U.S. where children and youth spend an average of 6-8 hours per day being sedentary [22-28]. Accumulating evidence shows that, independent of physical activity levels, sedentary behaviours are associated with increased risk of cardio-metabolic disease, all-cause mortality, and a variety of physiological and psychological problems [29-31]. Therefore, to maximize health benefits, approaches to resolve the inactivity crisis should attempt to both increase deliberate physical activity *and *decrease sedentary behaviours, especially in the pediatric population. However, to date, public health efforts have focused primarily on physical activity and have paid little attention to the mounting evidence to support sedentary behaviour as a distinct behaviour related to poor health.

A recent scoping review identified review articles, meta-analyses, and grey literature that examined the relationship between sedentary behaviour and health [32]. The large majority of this information reported on the relationship between screen time and body composition and did not include other indicators of health [23-25]. Furthermore, none of these reviews followed the rigorous process of a systematic review and are therefore not able to be used to inform the development of clinical practice guidelines. As a result, to our knowledge, there are no systematic, evidence-based sedentary behaviour guidelines for any age group, anywhere in the world. Guidelines that do exist are largely based on expert opinion or narrative literature reviews [33,34].

Therefore, the purpose of this systematic review was to gather, catalog, assess and evaluate the available evidence examining sedentary behaviours in relation to selected health outcomes in children and youth 5-17 years of age and present a summary of the best available evidence. Specifically, the review presents available evidence for minimal and optimal thresholds for daily sedentary time in children and youth, and when possible, how thresholds differ across health outcome or demographic status (i.e. age, gender). The information gathered in this review can serve to guide future research and inform the development of evidence-based clinical practice guideline recommendations for safe and healthy amounts of daily sedentary behaviour in the pediatric population.

## Methods

### Study Inclusion Criteria

The review sought to identify all studies that examined the relationship between sedentary behaviour and a specific health outcome in children and youth (aged 5-17 years). All study designs were eligible (e.g. cross sectional, retrospective, prospective, case control, randomized controlled trial (RCT), longitudinal). Longitudinal studies were included if the data presented in the article was consistent with the age limits that were set (i.e. if the study looked at participants at age 10 and then again at age 30, only baseline measurements from age 10 were used).

Studies were included only if there was a specific measure of sedentary behaviour. Eligible exposures of sedentary behaviours included those obtained via direct (e.g., measurements of sitting, or low activity measured by accelerometer) and self-reported (e.g., questionnaires asking about TV watching, video gaming, non-school computer use, and screen time - composite measures of TV, video games, computers) methods. Sedentary behaviour was often measured as a composite measure of all time engaging in sedentary behaviours including screen time outside of school hours. Six health indicators were chosen based on the literature, expert input, and a desire to have relevant measures from a range of holistic health indicators (i.e. not only physical health, but also emotional, mental and intellectual health). The six eligible indicators in this review were:

1. Body composition (overweight/obesity measured by body mass index (BMI), waist circumference, skin folds, bio-impedance analysis (BIA), dual-energy x-ray absorptiometry (DXA or DEXA));

2. Fitness (physical fitness, physical conditioning, musculoskeletal fitness, cardiovascular fitness);

3. Metabolic syndrome (MS) and cardiovascular disease (CVD) risk factors (unfavourable lipid levels, blood pressure, markers for insulin resistance or type 2 diabetes);

4. Self-esteem (self-concept, self-esteem, self efficacy);

5. Behavioural conduct/pro-social behaviour (child behaviour disorders, child development disorder, pro-social behaviour, behavioural conduct, aggression);

6. Academic achievement (school performance, grade-point average).

No Language or date limits were imposed in the search. The following definitions were used to help guide the systematic review [31]:

- *Sedentary: *A distinct class of behaviours (e.g. sitting, watching TV, playing video games) characterized by little physical movement and low energy expenditure (≤ 1.5 METs).

- *Sedentarism: *Engagement in sedentary behaviours characterized by minimal movement, low energy expenditure, and rest.

- *Physically active: *Meeting established physical activity guidelines (e.g. see Tremblay et al. 2011 for Canadian Physical Activity Guidelines [16]).

- *Physical inactivity: *The absence of physical activity, usually reflected as the proportion of time not engaged in physical activity of a pre-determined intensity and therefore not meeting established physical activity guidelines.

### Study Exclusion Criteria

As the volume of literature on sedentary behaviour was anticipated to be very high, to control the feasibility of this project, the following sample size limits were set *a priori*: population based studies (observational, cross sectional, cohort, and retrospective studies) were required to have a minimum sample size of 300 participants; RCTs, and intervention studies were required to have at least 30 participants. Studies of 'active gaming' (e.g., Nintendo Wii™, Microsoft Kinect™, Sony's Playstation Move™, video arcades, etc.) were excluded. Finally, studies that defined sedentary behaviour as 'failing to meet physical activity guidelines' were excluded from the review.

### Search strategy

The following electronic bibliographic databases were searched using a comprehensive search strategy to identify relevant studies: Ovid MEDLINE(R) (1950 to February Week 2 2010), Ovid EMBASE (1980 to 2010 Week 07), and Ovid psycINFO (1806 to February Week 3 2010). The search strategy was created by a single researcher (JM) and run by a second researcher (AL). The search strategies can be found in Additional file 1. The search was limited to studies looking at 'school-aged' children and youth (mean age of 5-17 years). Articles were extracted as text files from the OVID interface and imported in to Reference Manager Software (Thompson Reuters, San Francisco, CA). Duplicate articles were first removed using Reference Manager Software, and any remaining duplicates were removed manually. All articles were given a unique reference identification number in the database.

Titles and abstracts of potentially relevant articles were screened by two reviewers (AL and one of GG, MT, RC, RL or TS) and full text copies were obtained for all articles meeting initial screening by at least one reviewer. Two independent reviewers examined all full text articles (AL and one of GG, MT, RC, RL or TS) and any discrepancies were resolved by discussion and consensus between the two reviewers. If the reviewers were unable to reach consensus, a third reviewer was asked to look at the article in question. Consensus was obtained for all included articles.

Twelve key content experts were contacted and asked to identify the most influential papers from their personal libraries examining sedentary behaviour and health in the pediatric age group. Government documents from the U.S [6], the U.K. [17], and Australia [18] were used for reference and to help guide the review process.

### Data extraction

Standardized data extraction tables were created; data extraction was completed by one reviewer (AL) and checked by another (one of GG, RC, RL, or TS) for accuracy. Information was extracted regarding study characteristics (i.e. year, study design, country, number of participants, age), type of sedentary behaviour, measure of sedentary behaviour (i.e. direct, or indirect), and health outcome. Reviewers were not blinded to the authors or journals when extracting data.

### Risk of bias assessment

The Downs and Black checklist was used to asses study quality [35]. This 27 point checklist assesses the *quality of reporting *(e.g. "Are the main findings of the study clearly described"); *external validity *(e.g. "Were the subjects asked to participate representative of the entire population from which they were recruited"); *internal validity *(e.g. "Were subjects randomized to intervention groups"); and *power *(e.g. "Was there sufficient power such that the difference being due to chance is less than 5%"). The maximum score a study can receive is 32, with higher scores indicating better quality. Inter-rater reliability was calculated using Cohen's kappa.

Quality of evidence was determined by the study design and by Downs and Black score. Level of evidence was used to explain the quality of available studies and the confidence of the findings [36]. RCTs were considered to have the highest level of evidence while anecdotal reports were considered to have the lowest evidence. See Table 1 for more details. When possible, studies were examined for differences among age and gender subgroups.

**Table 1:** Criteria for assigning level of evidence to a recommendation

Level of evidence	Criteria
**Level 1**	- Randomized control trials without important limitations
**Level 2**	- Randomized control trials with important limitations - Observational studies (non-randomized clinical trials or cohort studies) with overwhelming evidence
**Level 3**	- Other observational studies (prospective cohort studies, case-control studies, case series)
**Level 4**	- Inadequate or no data in population of interest - Anecdotal evidence or clinical experience

Adapted from: Lau DC et al. 2007 [36]

### Analysis

A meta-analysis was performed with the data that were sufficiently homogeneous in terms of statistical, clinical, and methodological characteristics using Review Manager Software 5.0 (The Cochrane Collaboration, Copenhagen Denmark). Pooled estimates for the meta-analysis and their 95% confidence intervals were obtained using the random effects estimator of DerSimonian-Laird [37]. Studies were weighted by the inverse of their variance. Cochrane's Q was used to test for heterogeneity among studies and the I^2 ^(squared) index [10] was used to determine the degree of heterogeneity [38]. Funnel plots were used to assess publication bias (data not shown). Qualitative syntheses were conducted for remaining studies.

## Results

### Description of studies

**Figure 1:**
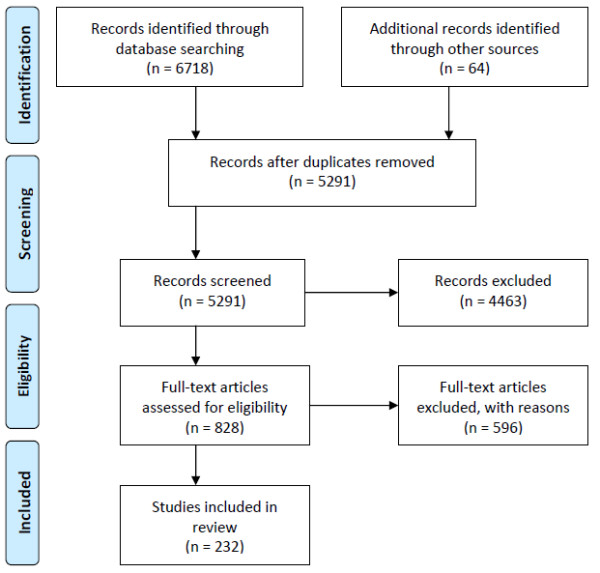
**Flow of information through the different phases of the review**.

After de-duplication, the preliminary search of electronic databases, reference lists, and grey literature identified 5,291 potentially relevant articles (Figure 1). Of these, 3,299 were identified in MEDLINE, 1,016 in EMBASE, 912 in psycINFO, and 64 through key informants, government documents, and bibliographies. After a preliminary review of titles and abstracts, 828 articles were included for detailed assessment of the full text article. Of these, 232 met the criteria for study inclusion (8 RCTs, 10 intervention studies, 37 longitudinal studies and 177 cross sectional studies). Individual study characteristics can be seen in Table 2. Reasons for excluding studies included: ineligible population (e.g. ineligible age or sample size) (n = 161), ineligible exposure (e.g. diet, physical activity) (n = 145), ineligible measure of sedentary behaviour (i.e. not meeting physical activity guidelines) (n = 19), ineligible outcome (n = 60), ineligible analysis (e.g. analysis focused on content of screen time versus duration of screen time, analysis focused on active video gaming) (n = 60), and 'other' (n = 216) (e.g. commentary article or methodological paper). Some studies were excluded for multiple reasons. Some articles (n = 9) could not be retrieved due to missing or incorrect reference information.

**Table 2:** Summary of characteristics of included studies

						*n *analyzed						
First Author	Year	Country	Grade	Age Range	Mean age	Total	Boys	Girls	Units of sedentary behaviour		Exposure	Outcome
RANDOMIZED CONTROLLED TRIALS												
Epstein LH [265]	1995	US		8-12	10.1	61			hour	week	TV	BC
Epstein LH [50]	2008	US		4-7	6	70	37	33	hour	day	TV	BC
Goldfield GS [264]	2006	Canada		8-12	10.4	30	13	17	min	day	TV	BC
Gortmaker SL [57]	1995	US			11.7	1295	668	627	hour	day	TV	BC
Hughes AR [262]	1991	Scotland		5-11	8.8	134	59	74	hour	day	SB	BC
Robinson TN [58]	1999	US				192			hour	week	TV, GAMES	BC
Robinson TN [221]	2003	US		8-10	9.5	61	0	61	hour	week	TV	BC, SE
Shelton D [263]	2007	Australia		3-10	7.5	43	20	23	hour	day	TV	BC
INTERVENTION STUDIES												
Epstein LH [56]	2000	US		8-12	10.5	76	24	52	hour	month	SB, ST	BC, FIT
Epstein LH [59]	2004	US		8-12	9.8	60	23	39	times	week	TV	BC
Epstein LH [60]	2005	US		8-16		58	28	30	hour	day	SB, TV	BC
Gentile DA [61]	2009	US			9.6	1323	685	674	hour	day	ST	BC
Goldfield GS [52]	2007	Canada		8-12	10.4	30	13	17	hour	day	SB	BC, SE
Harrison M [62]	2003	Ireland			10.2	312	177	135	min	day	TV, ST	BC
Ochoa MC [53]	2007	Spain		6-18	11.6	370	196	174	hour	week	TV	BC
Salmon J [51]	2008	Australia		1011	10.8	311	152	159	hour	day	TV	BC
Simon C [54]	2002	France			11.7	954	468	486	hour	day	TV, COMP	BC, SE
Tanasescu M [55]	2000	Puerto Rico		7-10	9.2	53	22	31	hour	day	TV	BC
LONGITUDINAL STUDIES										hour		
Aires L [83]	2010	Portugal		11-19		345	147	198	hour	day	SCREEN	BC, FIT
Berkey CS [76]	2003	US		10-15		11887	5120	6767	hour	day	TV, GAMES	BC
Bhargava A [77]	2008	US				7635			min	day	TV	BC
Blair NJ [68]	2007	England			5.5	591	287	304	hour	day	SB, TV	BC
Borradaile KE [86]	2008	US			11.2	1092	501	591	hour	week	TV	BC
Burke V [71]	2006	Australia			7.6/10.8	1569	630	648	hour	week	SCREEN	BC
Chen JL [78]	2007	Chinese		7-8	7.52	307	147	160	hour	day	TV, GAMES	BC
Danner FW [66]	2008	US				7334	3674	3660	hour	day	TV	BC
Dasgupta K [215]	2006	Canada			12.7/15.1/17.0	662	319	343	hour	week	SB, TV	MS
Day RS [85]	2009	US		8-14		556	277	279	min	day	TV	BC
Dietz WH [181]	1985	US		12-17		2153			hour	day	TV	BC
Elgar FJ [79]	2005	Wales			11.7	654	293	361	hour	week	TV	BC
Elgar FJ [79]	2005	Wales			15.3	392	181	211	hour	week	TV	BC
Ennemoser M [237]	2007	German		6-8		332			min	day	TV	SE, AA
Fulton JE [84]	2009	US		10-18		472	245	227	min	day	TV	BC
Gable S [70]	2007	US				8000			hour	day	TV	BC
Hancox RJ [88]	2004	New Zealand		5-15		1013			hour	day	TV	BC, MS
Hancox RJ [72]	2006	New Zealand		5-15		603	372	339	hour	day	SCREEN	BC
Henderson VR [67]	2007	US		11-19		2379	0	2379	hour	day	TV, SCREEN	BC
Hesketh K [80]	1997	Australia		5-10	7.6	1278	630	648	hour	day	SCREEN	BC
Hesketh K [80]	1997	Australia		8-13	10.7	1278	630	648	hour	day	SCREEN	BC
Hesketh K [64]	2009	Australia		5-10	7.7	1943	972	971	hour	day	TV, GAMES	BC
Hesketh K [64]	2009	Australia		8-13		1569	816	753	hour	day	TV, GAMES	BC
Jackson LA [223]	2009	US			12	500	235	265	hour	day	COMP, SCREEP	SE
Jago R [82]	2005	US		5-6	6.5	138	65	73	min	hr	SB, TV	BC
Janz KF [73]	2005	US			5.6/8.6	378	176	202	hour	day	SCREEN	BC
Johnson JG [41]	2007	US							hour	day	TV	AA
Kaur H [75]	2003	US		12-17		2223	1149	1074	hour	day	TV	BC
Lajunen HR [128]	2007	Finland		15-19		5184			hour		SB	BC
Lonner W [238]	1985	US		9-19	14.2	367			hour	day	TV	AA
Maffeis C [89]	1998	Italy			8.7	298	148	150	min	day	SCREEN	BC
Mistry K [229]	2007	US							hour	day	TV	PRO
Mitchell JA [49]	2009	UK		11-12	11.8	5434	2590	2844	hour	day	SB	BC, FIT
Must A [87]	2007	US		10-17		156	0	156	hour	day	SB, SCREEN	BC
O'Brien M [69]	2007	US		2-12		653			hour	week	TV	BC
Parsons TJ [74]	2005	England/Scotland/Wales			11/16	17733			hour	day	TV	BC
Purslow LR [63]	2008	England		8-9		345	176	169	min	day	SB	BC
Timperio A [65]	2008	Australia		10-12		344	152	192	times	week	SB, SCREEN	BC
Treuth MS [29]	2007	US			11.9	984	0	984	min	day	SB	BC
Treuth MS [27]	2009	US			13.9	984	0	984	min	day	SB	BC
Wosje,K.S [205]	2009	US		6.75-7.25		214			hour	day	SCREEN	FIT
CROSS SECTIONAL STUDIES												
Al SH [192]	2009	International		12-18		17715	8503	9212	hour	day	TV	BC
Albarwani S [207]	2009	Oman		15-16		529	245	284	hour	week	TV, COMP	FIT
Alves JG [191]	2009	Brazil		7-10		733	407	326	hour	day	TV	BC
Aman J [218]	2009	Sweden		11-18	14.5	2093	1016	991	hour	week	TV, COMP	MS
Andersen LF [155]	2005	Norway		8-14		1432	702	730	hour	day	TV	BC
Andersen RE [142]	1998	US		8-16		4063	1985	2071	hour	day	TV	BC
Anderson SE [103]	2008	US		4-12	8	2964	1509	1455	hour	day	TV	BC
Armstrong CA [213]	1998	US			9.28	588	304	284	hour	day	TV	FIT
Asante PA [183]	2009	US		3-13	8.5	324	182	142	hour	day	SCREEN	BC
Aucote HM [163]	2009	Australia	5-6		11.09	393	198	195	hour	week	TV, GAMES	BC
Barlow SE [151]	2007	US		6-17	12.1	52845			hour	day	TV	BC
Basaldua N [109]	2008	Mexico		6-12	8.9	551	278	273	hour	day	TV	BC
Bellisle F [123]	2007	France		9-11		1000	500	500	hour	day	TV	BC
Berkey CS [90]	2000	US			Sep-14	10769	4620	6149	hour	day	TV	BC
Beyerlein A [105]	2008	Germany		4.5-7.3		4967	2585	2382	hour	day	TV	BC
Boone JE [164]	2007	US			15.9	9155	4879	4276	hour	week	SCREEN	BC
Boone-Heinonen J [104]	2008	US		11-21		9251			hour		SB	BC
Boutelle KN [130]	2007	US		16-18		1726	890	836	hour	day	TV	BC
Brodersen NH [235]	2005	England			11.8	4320	2578	1742	hour	week	SB	SE, PRO
Bukara-Radujkovic G [96]	2009	Bosnia		11-12	11.5	1204	578	626	hour	day	TV, COMP	BC
Butte NF [119]	2007	US		6-17	10.8	897	441	456	hour	day	SCREEN	BC
Caldas S [245]	1999	US		4-19		34542			hour	day	TV	AA
Carvalhal MM [131]	2007	Portugal	10-11			3365	1755	1610	hour	day	TV, COMP	BC
Chaput J [154]	2006	Canada		5-10	6.6	422	211	211	hour	day	SCREEN	BC
Chen MY [78]	2007	Taiwan		13-18	15.03	660	351	309	hour	day	TV, COMP	BC, SE, PRO
Chowhan J [232]	2007	Canada		12-15		2666			hour	day	TV	PRO
Christoforidis A [95]	2009	Greece		4-18	11.41	1549	735	814	hour	day	SCREEN	BC, FIT
Collins AE [149]	2008	Indonesia		12-15		1758	815	916	hour	day	TV, COMP	BC
Colwell J [200]	2003	Japan		12-13		305	159	146	hour	day	SCREEN	BC, PRO
Cooper H [247]	1999	US	7-11			424	225	199	hour	day	TV	AA
Crespo CJ [177]	2001	US		8-16		4069	1994	2075	hour	day	TV	BC
Da CR [157]	2003	Brazil		7-10		446	107	107	hour	day	TV	BC
Dasgupta K [215]	2007	Canada		13-17		1267			hour	week	SCREEN	MS
Delva J [125]	2007	US				11265	5274	5991	hour	week	TV	BC
Dietz WH [181]	1985	US		12-17		6671			hour	day	TV	AA
Dietz WH [181]	1985	US		6-11		6965			hour	day	TV	BC, AA
Dollman J [211]	2006	Australia	6	10-11		843	439	404	min	Day	TV	FIT
Dumais SA [255]	2009	US		10-12		15850			hour		TV	AA
Dominick JR [225]	1984	US	10, 11	14-18		250	110	140	hour	Day	TV, GAME	SE, PRO
Eisenmann JC [175]	2002	US		14-18		15143			hour	day	TV	BC
Eisenmann JC [113]	2008	US'			16.2	12464	6080	6384	hour	day	TV	BC
Ekelund U [134]	2006	Europe		9-16		1921	911	1010	hour	day	TV	BC, MS
Fetler M [249]	1984	US	6			10603			hour	day	SCREEN	AA
Forshee RA [201]	2004	US		12-16	14	2216	1075	1141	hour	day	TV	BC
Forshee RA [188]	2009	US		5-18		1459	734	725	hour	week	SCREEN	BC
Gaddy GD [257]	1986	US				5074			hour	day	TV	AA
Giammattei J [140]	2003	US		11-14	12.6	385	186	199	hour	day	TV	BC
Gibson S [156]	2004	England		7-18		1294	655	639	min	day	TV	BC
Gomez LF [150]	2007	Colombia		5-12		11137	5539	5598	hour	day	TV, GAMES	BC
Gordon-Larsen P [176]	2002	US		11-19	15.9	12759	6290	6496	hour	week	TV, GAMES	BC
Gortmaker SL [143]	1996	US		10-15	11.5	746	388	358	hour	day	TV	BC
Gortmaker SL [57]	1999	US		6-11		1745			min	week	TV	SE, AA
Gortmaker SL [57]	1999	US		12-17		1745			min	week	TV	SE, AA
Graf C [167]	2004	Germany			6.8	344	177	167	hour	day	TV, COMP	BC
Grusser SM [40]	2005	Germany	6		11.83	323	175	148	hour	day	TV	AA
Hardy LL [133]	2006	Australia		11-15		2750	1446	1304	hour	day	SCREEN	FIT
Hernandez B [178]	1999	Mexico		9-16		461	244	217	hour	day	TV	BC
Hirschler V [144]	2009	Argentina		7-11	8.9	330	168	162	hour	day	TV	BC
Holder MD [222]	2009	Canada		8-12		375	252	262	hour	day	SCREEN	SE
Hume C [190]	2009	Netherlands			13	580	277	303	hour	day	SCREEN	BC
Islam-Zwart K [195]	2008	US				480	198	282	hour	day	TV	BC
Jackson LA [223]	2009	US			12.18	515	259	256	hour	day	GAMES, COMP	AA
Janssen I [166]	2004	Canada		11-16		5890	2812	3078	hour	day	TV, COMP	BC
Janz K [174]	2002	US		4-6	5.3	462	216	246	hour	day	TV	BC
Jaruratanasirikul S [241]	2009	Thailand	7-12		15.9	1492	562	929	hour		GAMES	AA
Johnson CC [41]	2007	US			12	1397	0	1397	hour	day	SB	SE
Katzmarzyk PT [197]	1998	Canada		9-18		784	423	361	min	day	TV	BC, FIT
Katzmarzyk PT [184]	1998	Canada				640	356	284	hour	day	TV	BC, FIT
Kautiainen S [135]	2005	Finland		14-18		6515	2916	3599	hour	day	SCREEN	BC
Keith TZ [256]	1986	US	high school seniors			28051			hour	day	TV	AA
Klein-Platat C [165]	2005	France			12	2714	1357	1357	hour	week	SB	BC
Kosti RI [196]	2007	Greece		12-17		2008	1021	987	hour	day	TV	BC
Kristjansson AL [243]	2009	Iceland		14-15		5810	2807	3004	hour	day	TV	AA
Kuntsche E [230]	2006	International		11-15		31177			hour	day	TV	PRO
Kuriyan R [117]	2007	India		6-16		598	324	274	hour	day	TV	BC
Lagiou A [160]	2008	Greece		10-12		633	316	317	hour	day	TV, GAMES	BC
Lajous M [92]	2009	Mexico		11-18	13.9	9132	3519	5613	hour	day	TV	BC
Lajunen HR [128]	2007	Finland			17.6	4098	1981	2117	hour	week	COMP	BC
Lasserre AM [116]	2007	Switzerland		10.1-14.9	12.3	5207	2621	2586	hour	day	TV	BC
Laurson KR [107]	2008	US		7-12		709	318	391	hour	week	SCREEN	BC
Lazarou C [217]	2009	Cyprus			11.7	622	306	316	hour	day	TV	MS
Leatherdale ST [11]	2008	Canada		14-19		25416	12806	12610	hour	day	TV	BC, PRO
Lioret S [127]	2007	France		3-14		1016	528	488	hour	day	SB, TV, COMP	BC
Lobelo F [208]	2009	US		14-18		5210	0	5210	hour	day	SCREEN	FIT
Lowry R [173]	2002	US				15349	7445	7828	hour	day	TV	BC
Lutfiyya MN [118]	2007	US		5-17		7972			hour	day	TV	BC
Maffeis C [114]	2008	Italy		8-10	9.3	1837	924	913	hour	day	TV	BC
Mark AE [220]	2008	US		12-19	15.9	1803	1005	798	hour	day	TV	BC, MS
McMurray RG [187]	2000	US		10-16	12.7	2389	1149	1240	hour	day	TV	BC
Mihas C [193]	2009	Greece		12-17	14.4	2008	1021	987	hour	day	SCREEN	BC
Mikolajczyk RT [194]	2008	Germany		11-17	13.5	4878	2433	2445	hour	low/high	SB	BC
Moraes SA [135]	2006	Mexico		6-14	8.0/11.3	662	343	339	hour	week		
Morgenstern M [94]	2009	Germany/US		10-17	12.8	4810	2294	2516	hour	day	SCREEN	BC
Morgenstern M [94]	2009	Germany/US		12-16	14	4473	2239	2234	hour	day	SCREEN	BC
Mota J [199]	2006	Portugal			14.6	450	220	230	hour	day	TV, COMP	BC
Muller MJ [179]	1999	Germany		5-7		1468	739	729	hour	day	TV	BC
Nagel G [193]	2009	Germany		6-9	7.57	1079		498	hour	day	TV, GAMES	BC
nastassea-Vlachou K [240]	1996	Greece		6-13		4690	2279	2411	hour	day	TV	AA
Nawal LM [148]	1998	US		5-18		62976			hour	day	TV, COMP	BC
Nelson MC [233]	2006	US		7-12		11957	5979	5978	hour	day	SCREEN	PRO
Neumark-Sztainer D [224]	2004	US		11-18	14.9	4746	2382	2364	hour	week	TV	SE, PRO
Nogueira JA [45]	2009	Brazil		8.3-16.8	13	326	204	122	hour	day	SB	BC
Obarzanek E [180]	1994	US		9-10	10.1	2379	0	2379	hour	week	TV	BC
Ohannessian CM [226]	2009	US		14-16	14.99	328	138	190	hour	day	SCREEN	SE, PRO, AA
Ortega FB [122]	2007	Spain		13-18.5	15.4	2859	1357	1502	hour	day	SB	BC
Overby NC [219]	2009	Norway		6-19		723	375	348	min	day	TV	
Ozmert E [42]	2002	Turkey				689	343	346	hour	day	TV	PRO, AA
Padez C [99]	2009	Portugal		7-9		3390	1696	1694	hour	day	TV	BC
Page RM [234]	2001	Philippine			15.1	3307	1267	1819	hour	week	TV	PRO
Pate RR [210]	2006	US		12-19	15.4	3287	1686	1601	hour	day	TV	FIT
Patrick K [169]	2004	US		11-15	12.7	878	407	471	min	day	TV	BC
Pratt C [101]	2008	US			12	1458	223	1235	hour	day	SB	BC
Purath J [185]	1995	US	3-5			365	189	176	hour	day	TV	BC, MS
Ramos E [126]	2007	Portugal		13		2161	1045	1116	min	week	SB, TV, COMP	BC
Rapp K [138]	2005	Germany			6.2	2140	1015	1125	hour	day	TV	BC
Ridley-Johnson R [252]	1983	US	5-8			290			hour	day	TV	AA
Roberts DF [250]	1984	US				539			hour	week	TV	AA
Robinson TN [58]	1999	US			12.4	971	0	971	hour	day	TV	BC
Ruangdaraganon N [141]	2002	Thailand		6-12	9.4	4197	2126	2035	hour	day	TV	BC
Russ SA [147]	2009	US		6-17		54863	28153	26710	hour	day	SCREEN	BC, SE
Sakamoto A [236]	1994	Japan	4-6			307	165	142	times	week	GAMES	PRO
Sakamoto A [236]	1994	Japan	4-6			537	287	250	hour	week	COMP, GAMES	PRO
Sakamoto A [236]	1994	Japan	4-5			118	118	0	hour	week	COMP, GAMES	PRO
Salmon J [136]	2006	Australia		5-12		1560	743	817	hour	day	TV	BC
Sardinha LB [48]	2008	Portugal		9-10	9.8	308	161	147	hour	day	SB	MS
Scott LF [254]	1958	US	6-7			407			hour		TV	AA
Sharif I [244]	2006	US		10-14		6522	3169	3353	hour	day	TV, GAMES	PRO, AA
Sharif I [260]	2010	US		9-15	12	4508	2209	2299	hour	day	TV, GAMES	AA
Shejwal B [246]	2006	India			16.05	654	368	286	hour	day	TV	AA
Shields M [162]	2006	US/Can		2-17		8661			hour	day	SB, TV	BC
Shin N [239]	2004	US		6-13	9	1203	605	598	min	day	TV	AA
Singh GK [106]	2003	US		10-17		46707	24072	22635	hour	day	TV	BC
Singh GK [106]	2003	US		10-17		46707	24072	22635	hour	day	TV	BC
Skoric MM [258]	2009	Singapore		8-12	10	333	180	153	hour		TV, GAMES	AA
Smith BJ [161]	2007	Fiji		11-16		443	200	245	hour	day	TV	BC
Spinks AB [124]	2007	Australia		5-12		518	282	236	min	week	SB, SCREEN	BC
Steffen LM [98]	2009	US		8-11		526	256	270	hour	day	TV	BC
Stettler N [168]	2004	Switzerland			8	872	410	462	hour	day	TV, GAMES	BC
Sugiyama T [47]	2007	US		12-19	15.9	4508	2295	2213	hour	day	SB	MS
Sun Y [91]	2009	Japan		12-13	.	5753	2842	2911	hour	day	TV	BC
Taylor WC [158]	2002	US		6-15	11.1	509	231	278	kcal	day	SB	BC
te Velde SJ [129]	2007	International		9-14	11.4	12538	6256	6282	hour	day	TV, COMP	BC
Thompson AM [189]	2009	Canada	3, 7, 11			1777	795	982	min	day	TV	BC
Toschke AM [112]	2008	Germany		5-6		4884			hour	day	TV	BC
Toschke AM [121]	2007	Germany		5-6		5472			hour	day	TV	BC
Trang NHHD [146]	2009	Australia		11-16		2660	1332	1328	hour	day	SCREEN	BC
Tremblay MS [172]	2003	Canada		7-11		7261			hour	day	TV	BC
Treuth MS [27]	2009	US		11-12	11.9	1579	0	1579	hour	day	SB	BC
Tsai H [153]	2007	Taiwan		11-12		2218	1146	1072	hour	day	TV	BC
Tsai H [145]	2009	Taiwan		11-12		1329	615	672	hour	day	SB, TV	BC
Tucker LA [212]	1987	US			15.7	406	406	0	hour	day	TV	FIT, SE, PRO
Tucker LA [206]	1986	US			15.7	379	379	0	hour	day	TV	FIT
Tucker LA [214]	1996	US		9-10	9.8	262	162	100	hour	day	TV	FIT
Ussher MH [231]	1007	England		13-16		2623			hour	day	TV	PRO, AA
Utter J [171]	2003	US			14.9	4480	2240	2240	hour	day	SCREEN	BC
Utter J [152]	2007	New Zealand		5-14		1743	959	784	hour	day	TV, COMP	BC
Vader AM [97]	2009	US			11, 7	11594	6162	5432	hour	day	TV	BC
van Schie EG [261]	1997	Netherlands		10-14	11.5	346	171	175	hour	day	SCREEN	PRO, AA
van Zutphen M [159]	2007	Australia		4-12	8	1926	939	987	min	day	TV	BC
Vandewater EA [170]	2004	US		1-12	6	2831	1444	1387	hour	day	SB, SCREEN	BC
Vaughan C [198]	2007	Australia		11-18	14	443	189	254	hour	day	SCREEN	BC
Vicente-Rodriguez G [110]	2008	Spain		13-18.5		1960	1012	948	hour	day	TV, GAMES	BC
Violante R [137]	2005	Mexico		6-14		8624	258	4366	hour	day	TV	BC
Wake M [186]	2003	Australia		5-13	9.1	2862	1445	1417	hour	week	SCREEN	BC
Walberg HJ [251]	1984	US	2-6		13	2890	1445	1445	hour	day	TV	AA
Walberg HJ [253]	1982	US			17	2001	1031	970	hour	day	TV	AA
Waller CE [202]	2003	China		6-11	9	880			hour	week	TV	BC
Wang Y [120]	2007	US			11.9	498	218	280	hour	day	SCREEN	BC
Welch WW [248]	1986	Australia	3-4	9	9	1960					TV	AA
Wells JC [108]	2008	Brazil		10-12		4452	2193	2258	hour	day	TV	BC, MS
Whitt-Glover MC [24]	2009	US		6-19		749	351	398	min	day	SB	BC
Wiggins J [227]	1987	US	4-12			483	252	231	min	day	TV	SE, AA
Wolf AM [203]	1998	US		11-14		552	0	552	hour	day	TV	BC
Wong SL [100]	2009	Canada			15.5	25060	12806	12254	hour	day	SB, SCREEN	BC
Zabinski MF [132]	2007	US		11-15		878	425	453	hour	day	SB	BC

SB, sedentary behaviour; TV, television viewing; COMP, computer time; GAME, video game playing; SCREEN, composite measure of 2 or more screen activities (i.e. television viewing, computer time, or video game playing); BC, body composition; MS, measures of metabolic syndrome and/or cardiovascular disease (e.g. insulin resistance, blood pressure); SE, self-esteem; PRO, pro-social behaviour; AA, academic achievement.

Table 2 provides a summary of all studies included in the review. The majority of the studies included in this systematic review were cross sectional (n = 177). In total, data from 983,840 participants were included in this review. Studies ranged from 30 participants in intervention studies and RCTs, to 62,876 participants in cross sectional observational investigations. Articles were published over a 51 year period from 1958 to 2009, and included participants ranging from 2-19 years of age. Although the scope of the review focused on those 5-17 years of age, studies that had a range below 5 years or over 17 years were not excluded as long as the mean age was between 5-17 years. Included studies involved participants from 39 countries; there were a greater number of articles reporting on female-only data than those reporting on male-only data. Translators were contracted to read non-English articles and complete any necessary data extraction for studies that met inclusion criteria (n = 8).

Of the 232 studies, 170 studies reported data on body composition, 15 on fitness, 11 on MS and CVD, 14 on self-esteem, 18 on pro-social behaviour, and 35 on academic achievement. The majority of studies (n = 223) used indirect measures to assess sedentary behaviour (i.e. parent-, teacher-, or self-report questionnaires). There were 14 studies [24,27,28,39-49] that directly measured sedentary behaviour with accelerometers and one that directly measured television viewing through a monitoring device [50]. The direction of the association between increased sedentary behaviour and health outcomes were similar between direct and indirect measures. Meta-analysis was conducted for RCTs examining change in body mass index.

### Risk of bias assessment

Risk of bias assessment was completed for all included studies (Additional file 2). The mean Downs and Black score was 20.7 (range = 16-26). The studies were then split into groups and labeled as 'high quality' (score 23-26, n = 36), 'moderate quality' (score 19-22, n = 169), and 'lower quality' (score 16-18, n = 27). Quality of study did not affect the outcome of the study; in other words, both lower quality and high quality studies showed a positive relationship between increased time spent sedentary and health risk. Inter-reviewer assessment using the Downs and Black tool was very high (kappa = 0.98).

### Data Synthesis

#### Body composition

Of the 232 studies included in this review, 170 examined body composition, with the majority of these focusing on the relationship between overweight and obesity and time spent watching TV (Table 3). Body composition was measured in a variety of ways including body mass index (BMI), sum of skin folds, percent body fat and various composite measures (e.g. BMI + sum of skin folds). Of the 8 RCTs, 7 showed that decreases in sedentary time lead to reductions in body weight (see meta-analysis below for details). Intervention studies reported desirable changes in body weight, BMI, and weight status among children and youth who successfully decreased their sedentary time [51-60]. Three intervention studies [61-63] reported that although sedentary behaviour decreased, there was no change in weight status (measured through BMI and skinfold thickness); however, these studies had relatively short follow-up periods (~1 year) and no control group leading the authors hypothesized that a longer follow up period was needed to detect a significant change in body composition. While nine-teen longitudinal studies reported that children who watched greater amounts of TV at baseline saw steeper increases in BMI, body weight and fat mass over time [64-82], nine longitudinal studies reported no significant relationship between time spent sedentary and weight status or fat mass [61-63,83-89]. Of the 119 cross sectional studies, 94 reported that increased sedentary time was associated with one or more of increased fat mass, increased BMI, increased weight status and increased risk for being overweight [28,90-182]. Risk for obesity increased in a dose response manner with increased time spent engaging in sedentary behaviours [92,106,110,128,156,178]. Twenty-five cross sectional studies reported no significant relationship between sedentary time and weight status [24,85,137,183-204]. One study [131] reported an effect in boys but not girls and one showed an effect in girls but not boys [139]. One study showed that among boys, being underweight was associated with more screen time [111]. The level of evidence reporting on the relationship between sedentary behaviour and body composition was of moderate quality and was classified as Level 2 with a mean Downs and Black score of 20.6 (standard deviation: ± 1.9).

**Table 3:** Summary table of results showing relation between sedentary behaviour and measures of body composition

Type of Study	Number of Studies	Number of participants	Narrative recommendation and main findings
RCT	8	1886	Reductions in sedentary behaviour are directly related to improved body composition.
Intervention	10	3547	TV watching and overweight/obesity were related in a dose-response manner (i.e. those who watched more TV were more likely to be overweight/obese).
Longitudinal	33	85753	TV watching and overweight/obesity were related in a dose-response manner (i.e. those who watched more TV were more likely to be overweight/obese).
Cross sectional	119	691759	> 2 hrs of sedentary behaviour related to increased risk of being overweight or obese.

Total of all studies	170	782884	Meta-analysis was performed on randomized controlled studies that looked at change in BMI. They found an effect of -0.89 kg/m^2 ^(95% CI of -1.67 to -0.11, p = 0.03) decrease in mean BMI in the intervention group. > 2 hrs of sedentary behaviour per day is associated with an increased risk for overweight/obesity. This risk increases in a dose-response manner. Each additional hour of TV viewing increased risk for obesity. > 2 hrs/day significantly increased risk for overweight/obesity. Mean Downs and Black score = 20.9 (± 1.9), Level 2 evidence.

#### Fitness

Fifteen studies assessed the relationship between time spent engaging in sedentary behaviour and fitness (Table 4). Increased time spent being sedentary was associated with decreased scores for overall physical fitness, VO_2 _max, cardiorespiratory fitness, and musculoskeletal fitness. An intervention reported that targeting decreased sedentary behaviour lead to increases in aerobic fitness [56]. This study (n = 13 boys and 26 girls, mean age = 10.5 years) showed that an intervention to decrease targeted sedentary behaviours (watching TV, playing computer games, talking on the telephone, or playing board games) led to increases in both physical activity and non-targeted sedentary behaviours. Longitudinal evidence was conflicting. One longitudinal study showed that > 2 hours per day of TV and computer use was associated with decreased musculoskeletal fitness [205]; while the second longitudinal study found no association between increased screen time and decreased fitness. Eight of 12 cross sectional studies showed that greater than 2 hours of screen time per day was associated with decreased VO_2_max, lower cardiorespiratory fitness, and lower aerobic fitness [95,206-212]. Two studies showed weak relationships between television watching and fitness [197,213]. Two studies showed no consistent association between television viewing and aerobic and musculoskeletal fitness [184,214]. The level of evidence related to fitness was classified as Level 3 with a mean Downs and Black score of 20.9 (standard deviation: ± 2.1), indicating moderate quality of reporting.

**Table 4:** Summary table of results showing relation between sedentary behaviour and fitness

Type of Study	Number of Studies	Number of participants	Narrative recommendation and main findings
RCT	0		
Intervention	1	76	Reductions in sedentary behaviour lead to increased fitness.
Longitudinal	2	561	One study showed no association whereas one study showed higher musculoskeletal fitness in those watching < 2 hrs of TV per day.
Cross sectional	12	17227	> 2 hrs of screen time per day is associated with better VO_2_max scores, better musculoskeletal and cardiorespiratory fitness scores.

Total of all studies	15	17864	Those watching less than 2 hours of TV a day showed higher results for fitness testing and more favourable bone health. Mean Downs and Black score = 20.6 (± 2.1), Level 3 evidence.

#### Metabolic syndrome and risk for cardiovascular disease

Eleven studies assessed the relationship between time spent engaging in sedentary behaviour and risk factors for MS and CVD (Table 5). All of the studies reported that increased sedentary time was associated with increased risk for MS or CVD. However, the results of these studies should be viewed with caution as the proportion of children and youth who have measurable health risk factors for MS or CVD is quite low. Longitudinal studies found that those watching more than 2 hours of television per day had higher serum cholesterol levels [88] and were more likely to have high blood pressure [215] than their peers who watched less TV. Cross sectional studies reported that high levels of screen time and self-reported sedentary behaviour were associated with increased risk for high systolic and diastolic blood pressure [47,108,216,217], higher HbA1 c [218], fasting insulin [134,216], insulin resistance [48,219], and MS [220]. These risk factors increase in a dose response manner with increased screen time [216,220]. One cross sectional study reported a significant relationship between watching TV and increased cholesterol in adolescents, but not in younger children [185]. The level of evidence for MS and CVD risk factors was classified as Level 3 with a mean Downs and Black score of 21.7 (standard deviation: ± 2.1), indicating moderate quality of reporting.

**Table 5:** Summary table of results showing relation between sedentary behaviour and markers for metabolic syndrome and cardiovascular disease

Type of Study	Number of Studies	Number of participants	Narrative recommendation and main findings
RCT	0		
Longitudinal	2	1675	> 2 hr of TV per day is associated with higher serum cholesterol levels. > 1.2 hrs of TV per day is associated with increased systolic blood pressure.
Cross sectional	9	17339	> 2 of screen time per day is associated with higher blood pressure and increased risk for metabolic syndrome.
Intervention	0		

Total of all studies	11	19014	Increased screen time is associated with increased risk for markers of metabolic syndrome and cardiovascular disease. Risk increases in a dose-response manner. Mean Downs and Black score = 21.7 (± 2.0), Level 3 evidence.

#### Self esteem

Fourteen studies assessed the relationship between time spent engaging in sedentary behaviour and self-esteem (Table 6). One RCT aimed to increase physical activity and decrease TV viewing [221], leading to a trend in improvements in self-esteem (P = 0.26) and concerns with body shape (p = 0.03). Intervention studies that targeted changes in sedentary behaviour produced inverse changes in physical self-worth and self-esteem [52,54]. Cross sectional studies showed that increased screen time was associated with higher depressive symptoms, low self-esteem, and decreased perceptions of self-worth [44,115,147,212,221-223]. There was evidence for a dose-response relationship as each additional hour of screen time seemed to increase the risk for lower self-esteem [147]. Two studies [224,225] reported that increased TV viewing was associated with decreased self-esteem in boys but not girls, and increased aggression in girls but not boys. Two studies showed no significant relationship [226,227]. One study [228] showed a significant relationship between increased TV viewing and decreased self-esteem in adolescents but not in young children. The level of evidence for studies examining self-esteem was classified as Level 3 with a mean Downs and Black score of 21.0 (standard deviation: ± 2.4) indicating moderate quality of reporting.

**Table 6:** Summary table of results showing relation between sedentary behaviour and self-esteem

Type of Study	Number of Studies	Number of participants	Narrative recommendation and main findings
RCT	1	61	Girls who decreased sedentary behaviour had lower body dissatisfaction and showed a trend towards improved self-esteem.
Intervention	2	984	Decreases in sedentary behaviour lead to improved self worth and self-esteem.
Longitudinal	0		
Cross sectional	11	71068	Those with higher reported sedentary behaviour had poorer scores on self worth. This association seems to increase in a dose-response manner

Total of all studies	14	72113	Each additional hour of TV viewing was associated with decreases in self-worth and self-concept. Mean Downs and Black score = 21.0 (± 2.4), Level 3 evidence.

#### Pro-social behaviour

Eighteen studies assessed the relationship between time spent engaging in sedentary behaviour and pro-social behaviour (Table 7). The one longitudinal study examining the relationship between sedentary behaviour and pro-social behaviour found that sustained TV exposure (i.e. ≥ 2 hours per day) was a significant risk factor for behavioural problems [229]. Cross sectional studies reported similar findings. Those who watched less TV were more emotionally stable, sensitive, imaginative, outgoing, self-controlled, intelligent, moralistic, college bound, and less likely to be aggressive or to engage in risky behaviour [42,115,230-235]. Two studies found a significant relationship between increased computer use and behaviour problems in boys [111,236] but not girls. One study showed that increased TV viewing was associated with aggression in girls but not boys [225]. The level of evidence for studies reporting on pro-social behaviour was classified as Level 3 with a mean Downs and Black score of 19.9 (standard deviation: ± 1.3) indicating moderate quality of reporting.

**Table 7:** Summary table of results showing relation between sedentary behaviour and pro-social behaviour

Type of Study	Number of Studies	Number of participants	Narrative recommendation and main findings
RCT	0		
Longitudinal	1	2707	Watching > 2 hrs of TV per day is a risk factor for social behaviour problems
Intervention	0		
Cross sectional	17	91934	Individuals watching > 3 hrs of TV per day are more likely to exhibit poor social behaviours and be more aggressive. Limited evidence to suggest this relationship is stronger in boys.

Total of all studies	18	94391	> 2 hrs of TV per day is associated with poor pro-social behaviour. Those watching less than 3 hrs of TV per day scored more positively in aspects of pro-social behaviour Mean Downs and Black score = 19.9 (± 1.34), Level 3 evidence.

#### Academic achievement

Thirty five studies assessed the relation between time spent engaging in sedentary behaviour and academic achievement (Table 8). Academic achievement was measured in a variety of ways but included measures of I.Q., school grades, grade point average (GPA), performance on standardized tests, and self-report questionnaires (e.g. students rated their own level of academic achievement). The longitudinal studies included in this review found that children who watched higher amounts of TV had greater difficulties with attention as teenagers [41], showed lower progression for reading level [237], and performed worse on cognitive tests [238] than those watching less than one hour of television per day. The majority of cross sectional studies (75%) reported that children and youth who watched higher levels of TV tended to spend less time doing homework, studying, and reading for leisure which may lead to a decrease in academic achievement [42,181,239-255]. This association increased in a dose response manner [181,244,248]. Ten of the cross sectional studies found no significant relationship [57,226,227,238,256-261]. One study [228] found that this relationship was significant in adolescents but not younger children. The evidence for academic achievement was classified as Level 3 with a mean Downs and Black score of 19.2 (standard deviation: ± 2.1) indicating moderate quality of reporting.

**Table 8:** Summary table of results showing relation between sedentary behaviour and academic achievement

Type of Study	Number of Studies	Number of participants	Narrative recommendation and main findings
RCT	0		
Longitudinal	3	3530	Watching > 1 hr of TV per day is associated with attention difficulties.
Intervention	0		
Cross sectional	32	157637	> 2 hrs of screen time per day resulted in lower academic achievement.
Intervention	0		

Total of all studies	35	161167	> 2 hrs of screen time per day is negatively associated with academic achievement. Dose-response relation between time spent playing video games, watching TV and using the computer (for non-academic purposes). > 3 hrs/day associated with poor school performance and lower I.Q. scores. Mean Downs and Black score = 19.1 (± 2.1), Level 3 evidence.

#### Quantitative data synthesis

Data for each of the outcomes were assessed to determine if they were sufficiently homogeneous to make meta-analysis appropriate. The only outcome for which data were consistently collected and reported and for which the characteristics of the studies were similar enough to undertake a meta-analysis was body composition. However, this was only for the RCTs; the longitudinal, cross sectional and intervention studies that examined body composition had too many inconsistencies to allow for a quantitative synthesis of results.

**Figure 2:**
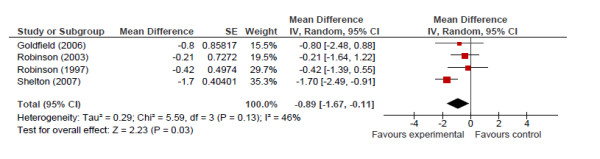
**Meta-analysis of randomized controlled studies examining decreases in sedentary behaviour and effect on body mass index**.

Change in mean BMI before and after the intervention (at the longest point of follow-up for each study) was used as the point estimate for the meta-analysis of the RCT data. Of the 8 RCTs, only 6 had data that could be used to calculate the change in BMI after the intervention [50,58,221,262-264] (the other two reported on prevalence of overweight and obesity) [57,265]. Of the remaining six studies, one [50] examined standardized estimates of BMI only and one [262] presented only median change in BMI and not a mean change. Study authors were contacted for missing information, but no additional data was made available and thus these studies were excluded from the meta-analysis. Meta-analysis of the 4 RCTs that remained revealed an overall significant effect of -0.89 kg/m^2 ^(95% CI of -1.67 to -0.11, p = 0.03) indicating an overall decrease in mean BMI associated with the interventions (Figure 2). The Chi square test for heterogeneity was not significant but the I^2 ^was 46% indicating that there was low to moderate heterogeneity in the data. The funnel plot showed no indication of publication bias (data not shown).

Meta-analyses were not undertaken for other outcomes or study designs because there was substantial heterogeneity in the units of measures and type of reporting of sedentary behaviour, as well as the specific measures of each outcome. For example, when reporting on the relation between time spent watching TV and overweight and obesity, one study may report the relation between the frequency of TV watching and skin fold thickness, whereas another may examine the relation of daily volume of TV watching and BMI. Even for studies that examined the same outcome, for instance BMI, some would report the proportion overweight or obese, while others would report mean BMI. In addition, some studies reported on data for males or females only, while others reported only overall estimates and many were missing key information about participant characteristics or study design. As a result, we were unable to determine common point estimates and associated measures of errors for many of the studies. Due to the scope of the review, it was not feasible to contact every author for individual data to re-run the analyses. Developing reporting standards for primary studies examining the relationship between sedentary behaviour and health would help to ensure that appropriate data are available for future meta-analyses.

## Discussion

Based on this systematic review of 232 studies, sedentary behaviour (assessed primarily through increased TV viewing) for more than 2 hours per day was associated with unfavourable body composition, decreased fitness, lowered scores for self-esteem and pro-social behaviour and decreased academic achievement in school-aged children and youth (5-17 years). This was true for all study designs, across all countries, using both direct and indirect measurements, and regardless of participant sample size. All studies examining risk factors for MS and CVD disease reported that increased sedentary time was associated with increased health risk; however, the included studies examined a wide range of risk factors, and thus there was insufficient evidence to draw conclusions on the relationship for metabolic risk as a whole.

High heterogeneity of the included studies limited meta-analysis to RCTs examining the relationship between television viewing and BMI. This revealed a trend to support the hypothesis that decreased time spent sedentary is associated with decreases in BMI. This result should be interpreted cautiously, given that it is only based on a small number of RCTs and that only half of the RCTs included in the review were included in the meta-analysis. Nonetheless, this meta-analysis of RCTs, which are considered to be the highest quality of research evidence, coupled with the qualitative syntheses of data from the other study designs, provides consistent evidence of the inverse relationship between sedentary behaviour and health outcomes, and that reducing sedentary behaviour can improve body composition. Furthermore, this finding was consistent with the results of observational studies and previous reviews [19-21,23,25].

Studies included in this review used primarily indirect measures (i.e. parent, teacher, and self-report questionnaires) to assess time spent engaging in sedentary behaviour. Those studies that did use direct (i.e. accelerometer) measures found that children and youth are spending a large proportion of their day (up to 9 hours) being sedentary [24,27,29,39-47,49,178]. Therefore, for some children and youth, a viable approach to improving health may be to work towards a reduction of at least *some *of their sedentary behaviours either through smaller, micro-interventions (e.g. interrupting prolonged sedentary time), or lager macro-interventions (e.g. population-based interventions and public health initiatives). Decreasing sedentary time is important for all children and youth, but it may be may be especially important to promote gradual decreases in the *most *sedentary group as a stepping stone to meeting sedentary behaviour guidelines [266].

### Strengths and limitations

Strengths of this review included a comprehensive search strategy, *a-priori *inclusion and exclusion criteria and analyses, and inclusion of non-English language articles. We included direct and indirect measures of sedentary behaviour and focused on 6 diverse health indicators in children and youth. Although efforts were made to include grey literature (e.g. by contacting key informants and reviewing government documents), we did not include conference proceedings and other types of grey literature because it was impractical and unfeasible to sift through all unpublished work, and also because of limitations in the quality of reporting in conference abstracts [267,268]. We do not anticipate that additional, unpublished work would change the results.

Our study has limitations, including the types of outcome measurements and analyses reported in the primary studies and primary study quality. The scope of this review was large and included a great deal of health indicators and measurement tools. A more detailed meta-analysis would have allowed us to estimate the overall effect sizes for each outcome. However, due to the heterogeneity of the data, it was impossible to complete such analysis. Furthermore, some studies had missing information on participant characteristics making it impossible to determine if basic demographics act as a confounder for the relationship between sedentary behaviour and health. Many studies also grouped their variables into tertiles, or groups that also took into account physical activity level. Although it was still possible to ascertain information regarding the association between level of sedentary behaviour and health indicators, it made it very difficult to compare the information across studies. Similarly, very few studies measured time spent being sedentary directly (i.e. with direct observation or accelerometry). Previous work [269,270] has shown significant differences between direct and indirect measures of physical activity; similar work needs to be completed with respect to sedentary behaviour to gain a better understanding of possible biases in previous studies. Indirect measurements of sedentary behaviour often lead to grouping for analyses. This may lead to bias in the results of the systematic review as many studies arbitrarily grouped their participants as ''high users" if they watched more than 2 hours of television per day. This could perhaps be falsely leading us to conclude that 2 hours is the critical cut-point or threshold. Further work using direct (i.e. accelerometer) measures of sedentary behaviour and screen time as continuous variables will help to clarify if a cut-point of 2 hours is in fact biased.

The final important limitation of this review was the type of primary studies that were available for analysis. Studies with small sample sizes were excluded; however we do not believe that this had a significant impact upon the strength or direction of associations observed in this review. The majority of studies (78.4%) included in this review were cross sectional, observational studies, using indirect (i.e. parent-, teacher, or self-report) measurements of sedentary behaviour. Cross sectional data make it impossible to infer causation and results should therefore be interpreted with caution. However, it should be noted that due to ethical considerations, it may be impossible to conduct a RCT on the effects of long periods of sedentary behaviours in children and youth. Due to the large and diverse sample sizes available in population-based cross sectional research, and given that this information demonstrates similar trends as those seen in RCTs and intervention studies, we believe that the evidence presented in this review provides important insights into the relationship between sedentary behaviour and health outcomes in school-aged children and youth.

### Future work

The purpose of this review was to provide an evidence base to inform clinical practice sedentary behaviour guidelines for children and youth [266]. Future work is needed to translate this information into clinical practice guidelines and disseminate this information to health care providers and the general public. While this review was limited to children and youth, similar work is needed to inform sedentary guidelines for young children aged 0-5 years, adults, and older adults.

As the accessibility and popularity of multiple forms of screen-based technology increases among the pediatric population, future work needs to continue to focus on media engagement. Specifically, with increasing popularity for hand-held, portable devices, 'sedentary multitasking' is becoming increasingly common. Children and youth are able to watch television, talk on the phone, and use the computer at the same time. This is a relatively new phenomenon and we are currently unaware what, if any, are the health effects associated with this high level of 'multi-screen' time. This is also true for the effect of advancements in technology and their associated health effects. For example, 'active video gaming' (e.g., Nintendo Wii™, Microsoft Kinect™, Sony's Playstation Move™) is advertised as an effective mode of physical activity. Although it is true that some games can require sufficient energy expenditure for health benefits [271], the socio-cognitive and physiological aspects of remaining indoors for long periods are unknown. Furthermore, children and youth can learn quite quickly how to use minimal gestures (e.g., using wrist movement only) to play the game thereby substantially reducing energy expenditure.

Finally, as described above, the vast majority of the current evidence has been based on self-report questionnaires focused on TV viewing and body composition. It is now clear that these two variables are related. Future work needs to move beyond this relationship and focus on other modes of sedentarism (e.g., prolonged sitting, passive transport) and other associated health indicators. To do this, objective measures of the time, type and context of sedentary pursuits will be needed in combination with robust and standardized measures of health indicators.

## Conclusions

Physical inactivity and sedentary behaviour are pervasive and persistent public health challenges to overcome. This review demonstrates that there is a need to advocate for increases in physical activity AND decreases in sedentary behaviour. It is believed that a multi-level, multi-sectoral approach is required for this to be successful [11]. Ultimately, resolving the problem of inactivity requires a sustained change in individual daily activity and sedentary patterns. From a public health perspective, a reduction in sedentary behaviour may be easier than increasing physical activity *per se *because there are fewer restrictions (i.e. no need to change clothing or use special equipment), and can be easily attained with minimal burden to a person's time or financial resources.

This systematic review summarizes the current evidence examining the relationship between sedentary behaviours and a series of health indicators. It was determined that increased sedentary time was associated with negative health outcomes in both boys and girls; this was true across all study designs with the majority of studies (85.8%) reporting similar relationships. The majority of current work has focused on television viewing and body composition and suggests that children and youth should watch less than 2 hours of TV per day during their discretionary time. Furthermore, children and youth should try to minimize the time they spend engaging in other sedentary pursuits throughout the day (e.g. playing video games, using the computer for non-school work or prolonged sitting). This work can be used to inform the development of evidence-based sedentary behaviour recommendations for children and youth.

## List of Abbreviations

BMI: Body Mass Index; CVD: Cardiovascular disease; DXA or DEXA: Dual-energy x-ray absorptiometry; MS: Metabolic syndrome; RCT: Randomized controlled trial; TV: Television.

## Competing interests

All authors received partial financial support from the Public Health Agency of Canada; no other competing interests exist.

## Authors' contributions

MT was responsible for the initiation, conceptualization and design of the systematic review; oversaw the data collection and extraction, analysis, and interpretation of data and was responsible for revising the manuscript critically for important intellectual content. AL was responsible for conducting the search, data collection and extraction, the risk of bias assessment, analysis and interpretation of data, and drafting the manuscript. MEK was responsible for the design and methodology of the review and revising the manuscript critically for important intellectual content. SCG was responsible for the design and methodology of the manuscript, conducting the meta-analysis, and revising the manuscript critically for important intellectual content. RC, GG, TS and RL were responsible for data collection and extraction, risk of bias assessment, and were responsible for revising the manuscript critically for important intellectual content. JM was responsible for the generation of systematic review search terms. MS was responsible for methodology of the review. All authors have read and approved the final manuscript. MT is the guarantor of the paper.

## Supplementary Material

Additional file 1 Search strategy. Click here for file

Additional file 2 Search strategy. Click here for file
