# Supplementary material for: Systematic review of sedentary behaviour and health indicators in school-aged children and youth
Source: Int J Behav Nutr Phys Act. 2011 Sep 21;8:98. doi: 10.1186/1479-5868-8-98 (PMC3186735; doi:10.1186/1479-5868-8-98)
Supplement: Additional file 1 — Search strategy. [file 1479-5868-8-98-S1.DOC]

**MEDLINE Search Strategy**

Completed on February 23^rd^, 2010

| 1. sedentar$.tw. |
| --- |
| 2. Sedentary Lifestyle/ |
| 3. ((chair or sitting or car or automobile or auto or bus or indoor or in-door or screen or computer) adj time).tw. |
| 4. low energy expenditure.tw. |
| 5. (computer game* or video game* or ((television adj watch*) or tv watch*)).tw. |
| *6. television/ or computers/ or video games/* |
| *7. (screen based entertainment or screen-based entertainment or screen time).tw.* |
| *8. physical inactivit*.tw.* |
| 9. bed rest.mp. |
| 10. sitting.tw. |
| **11. or/1-10** |
| 12. exp obesity/ |
| 13. (obesit* or obese).tw. |
| 14. exp overweight/ |
| 15. (overweight or over-weight).tw. |
| 16. exp Body Fat Distribution/ |
| 17. exp body composition/ |
| 18. Waist Circumference/ |
| 19. waist circumference.tw. |
| 20. Skinfold Thickness/ |
| 21. (skin folds or skin-fold*).tw. |
| 22. (body composition* or BMI or body mass index).tw. |
| 23. exp "body weights and measures"/ |
| 24. (bio-impedance analysis or BIA).tw. |
| 25. Absorptiometry, Photon/ |
| 26. (absorptiometery or densitometry or photodensitometry or DXA or DEXA).tw. |
| 27. Physical Fitness/ |
| 28. (physical conditioning or fitness).tw. |
| 29. musculoskeletal fitness.tw. |
| 30. cardiovascular fitness.tw. |
| 31. metabolic syndrome x/ |
| 32. Insulin Resistance/ |
| 33. (metabolic cardiovascular syndrome or metabolic syndrome or syndrome x).tw. |
| 34. exp cardiovascular diseases/ |
| 35. risk factors/ |
| 36. 33 and 34 |
| 37. ((cardiovascular disease$ or heart disease$ or vascular disease$) adj risk$).tw. |
| 38. exp self concept/ |
| 39. (self-esteem or self esteem).tw. |
| 40. exp child development disorders/ |
| 41. child behavio?r disorders/ |
| 42. (pro-social behavio?r or prosocial behavio?r or pro social behavio?r).tw. |
| 43. exp *social behavior/ |
| 44. (behavio?ral conduct or behavio?ral conduct).tw. |
| 45. (academic achievement or educational achievement).tw. |
| 46. educational achievement/ |
| 47. School Admission Criteria/ |
| 48. (grade-point average or grade point average or GPA).tw. |
| 49. or/12-33 |
| 50. or/36-49 |
| 51. 11 and 50 |
| 52. limit 51 to ("child (6 to 12 years)" or "adolescent (13 to 18 years)") |
| 53. (child* or adolescent* or youth* or pediatric* or paediatric*).tw. |
| 54. 51 and 53 |
| 55. 52 or 54 |

**Embase Search Strategy**

Completed on February 23^rd^, 2010

|  |
| --- |
| 1. sedentar$.tw. |
| 2. ((chair or sitting or car or auto or automobile or bus or indoor or in-door or screen or computer) adj time).tw. |
| 3. low energy expenditure.tw. |
| 4. (computer game* or video game*).tw. |
| 5. ((television adj watch*) or tv watch*).tw. |
| 6. (screen based entertainment or screen-based entertainment or screen time).tw. |
| 7. television viewing/ or computer/ or recreation/ |
| 8. sitting.tw. |
| 9. bed rest.mp. |
| 10. physical inactivit*.tw. |
| 11. or/1-10 |
| 12. (obesit* or overweight or obese or over-weight).tw. |
| 13. exp obesity/ |
| 14. exp body weight/ |
| 15. exp Body Fat Distribution/ |
| 16. (body composition or BMI or body mass index).tw. |
| 17. waist circumference.tw. |
| 18. Waist Circumference/ |
| 19. Skinfold Thickness/ |
| 20. (skin fold* or skin-folds).tw. |
| 21. (bio-impedance analysis or bio impedance analysis or BIA).tw. |
| 22. (absorptiometery or densitometry or photodensitometry or DXA or DEXA).tw. |
| 23. photon absorptiometry/ |
| 24. fitness/ |
| 25. (physical conditioning or fitness).tw. |
| 26. musculoskeletal fitness.tw. |
| 27. metabolic syndrome x/ |
| 28. (metabolic cardiovascular syndrome or metabolic syndrome or syndrome x).tw. |
| 29. exp cardiovascular disease/ |
| 30. risk factor/ |
| 31. 29 and 30 |
| 32. ((cardiovascular disease$ or heart disease$ or vascular disease$) adj risk$).tw. |
| 33. exp child development/ |
| 34. developmental disorder/ |
| 35. behavio?ral conduct.tw. |
| 36. exp *social behavior/ |
| 37. (pro-social behavio?r or prosocial behavio?r or pro social behavio?r).tw. |
| 38. exp self concept/ |
| 39. self-esteem.tw. |
| 40. (academic achievement or educational achievement).tw. |
| 41. exp academic achievement/ |
| 42. (grade-point average or grade point average or GPA).tw. |
| 43. or/12-28 |
| 44. or/31-43 |
| 45. 11 and 44 |
| 46. limit 45 to (school child <7 to 12 years> or adolescent <13 to 17 years>) |
| 47. (child* or youth* or adolescent* or pediactric* or paediatric*).tw. |
| 48. 45 and 47 |
| 49. 46 or 48 |

**PsycINFO Search Strategy**

Completed on February 23^rd^, 2010

|  |
| --- |
| 1. sedentar*.tw. |
| 2. ((chair or sitting or car or automobile or auto or bus or indoor or in-door or screen or computer) adj time).tw. |
| 3. (computer game* or video game*).tw. |
| 4. ((television adj watch*) or tv watch*).tw. |
| 5. (screen based entertainment or screen-based entertainment or screen time).tw. |
| 6. television viewing/ or computers/ or computer games/ |
| 7. physical inactivit*.tw. |
| 8. bed rest.tw. |
| 9. sitting.tw. |
| 10. low energy expenditure.tw. |
| 11. or/1-10 |
| 12. exp obesity/ |
| 13. exp body weight/ or Body Mass Index/ |
| 14. (obesit* or obese or overweight or over-weight).tw. |
| 15. (body composition* or body mass index or BMI).tw. |
| 16. waist circumference.tw. |
| 17. (skin fold* or skin-fold*).tw. |
| 18. (bio impedance analysis or BIA).tw. |
| 19. (absorptiometery or densitometry or photodensitometry or DXA or DEXA).tw. |
| 20. exp physical fitness/ |
| 21. (physical conditioning or fitness).tw. |
| 22. musculoskeletal fitness.tw. |
| 23. cardiovascular fitness.tw. |
| 24. exp cardiovascular disorders/ |
| 25. risk factors/ |
| 26. 24 and 25 |
| 27. ((cardiovascular disease$ or heart disease$ or vascular disease$) adj risk$).tw. |
| 28. (metabolic cardiovascular syndrome or metabolic syndrome or syndome x).tw. |
| 29. exp metabolic syndrome x/ |
| 30. exp cognitive development/ |
| 31. ((cognit* adj development) or (behavio?ral conduct or behavio?ral conduct)).tw. |
| 32. exp bahavior problems/ or exp behavior disorder/ or conduct disorder/ |
| 33. (pro-social behavio?r or prosocial behavio?r or pro social behavio?r).tw. |
| 34. exp prosocial behavior/ |
| 35. exp self-concept/ |
| 36. exp self-esteem/ |
| 37. (self esteem or self-esteem).tw. |
| 38. (academic achievement or educational achievement).tw. |
| 39. (grade-point average or grade point average or GPA).tw. |
| 40. exp academic achievement/ or Academic Achievement Motivation/ or academic self concept/ |
| 41. exp behavio?r problems/ or exp behavio?r disorder/ or conduct disorder/ |
| 42. or/12-23 |
| 43. or/26-42 |
| 44. 11 and 43 |
| 45. limit 44 to (180 school age or 200 adolescence ) |
| 46. (child* or youth* or adolescent* or pediatric* or paediatric*).tw. |
| 47. 44 and 46 |
| 48. 45 or 47 |
