# Supplementary material for: Systematic review of sedentary behaviour and health indicators in school-aged children and youth
Source: Int J Behav Nutr Phys Act. 2011 Sep 21;8:98. doi: 10.1186/1479-5868-8-98 (PMC3186735; doi:10.1186/1479-5868-8-98)
Supplement: Additional file 2 — Search strategy. [file 1479-5868-8-98-S2.DOC]

| **Author** | **year** | **Reporting (_/11)** | **External Validity (_/3)** | **Internal Validity**  **– Confounding (_/7)** | **Internal Validity**  **– Bias (_/6)** | **Power (_/5)** | **Total score**  **(_/32)** |
| --- | --- | --- | --- | --- | --- | --- | --- |
| **Randomized Controlled Studies** |  |  |  |  |  |  |  |
| Epstein LH^265^ | 1995 | 10 | 3 | 5 | 1 | 5 | 24 |
| Epstein LH^50^ | 2008 | 7 | 3 | 5 | 1 | 5 | 21 |
| Goldfield GS^264^ | 2006 | 6 | 2 | 5 | 3 | 5 | 21 |
| Gortmaker SL^57^ | 1995 | 8 | 3 | 5 | 5 | 5 | 26 |
| Hughes AR^262^ | 1991 | 7 | 3 | 5 | 1 | 5 | 21 |
| Robinson TN^58^ | 1999 | 7 | 3 | 5 | 2 | 5 | 22 |
| Robinson TN^221^ | 2003 | 10 | 3 | 5 | 3 | 5 | 26 |
| Shelton D^263^ | 2007 | 7 | 3 | 5 | 1 | 5 | 21 |
| **Intervention** |  |  |  |  |  |  |  |
| Epstein LH^56^ | 2000 | 9 | 3 | 5 | 2 | 5 | 24 |
| Epstein LH^59^ | 2004 | 7 | 3 | 5 | 1 | 5 | 21 |
| Epstein LH^60^ | 2005 | 6 | 3 | 4 | 1 | 5 | 19 |
| Gentile DA^61^ | 2009 | 5 | 3 | 5 | 1 | 5 | 19 |
| Goldfield GS^52^ | 2007 | 7 | 3 | 5 | 1 | 5 | 21 |
| Harrison M^62^ | 2003 | 7 | 3 | 5 | 1 | 5 | 21 |
| Ochoa MC^53^ | 2007 | 8 | 3 | 3 | 2 | 5 | 21 |
| Salmon J^51^ | 2008 | 4 | 3 | 5 | 1 | 5 | 18 |
| Simon C^54^ | 2002 | 7 | 3 | 5 | 2 | 5 | 22 |
| Tanasescu M^55^ | 2000 | 6 | 3 | 5 | 2 | 5 | 21 |
| **Longitudinal** |  |  |  |  |  |  |  |
| Aires L^83^ | 2010 | 8 | 3 | 5 | 2 | 5 | 23 |
| Berkey CS^76^ | 2003 | 4 | 3 | 5 | 1 | 5 | 18 |
| Bhargava A^77^ | 2008 | 5 | 3 | 5 | 1 | 5 | 19 |
| Blair NJ^68^ | 2007 | 7 | 3 | 5 | 1 | 5 | 21 |
| Borradaile KE^86^ | 2008 | 5 | 3 | 5 | 1 | 5 | 19 |
| Burke V^71^ | 2006 | 8 | 3 | 5 | 1 | 5 | 22 |
| Chen JL^78^ | 2007 | 5 | 3 | 5 | 1 | 5 | 19 |
| Danner FW^66^ | 2008 | 9 | 3 | 5 | 2 | 5 | 24 |
| Dasgupta K^215^ | 2006 | 6 | 3 | 5 | 1 | 5 | 20 |
| Day RS^85^ | 2009 | 6 | 3 | 5 | 2 | 5 | 21 |
| Dietz WH^181^ | 1985 | 7 | 3 | 5 | 1 | 5 | 21 |
| Elgar FJ^79^ | 2005 | 6 | 3 | 5 | 1 | 5 | 20 |
| Ennemoser M^237^ | 2007 | 5 | 3 | 5 | 1 | 5 | 19 |
| Fulton JE^84^ | 2009 | 8 | 3 | 5 | 2 | 5 | 23 |
| Gable S^70^ | 2007 | 8 | 3 | 5 | 2 | 5 | 23 |
| Hancox RJ^88^ | 2004 | 7 | 3 | 5 | 1 | 5 | 21 |
| Hancox RJ^72^ | 2006 | 7 | 3 | 5 | 1 | 5 | 21 |
| Henderson VR^67^ | 2009 | 5 | 1 | 3 | 2 | 5 | 16 |
| Hesketh K^80^ | 1997 | 7 | 2 | 5 | 1 | 5 | 20 |
| Hesketh K^64^ | 2009 | 5 | 3 | 5 | 1 | 5 | 19 |
| Jackson LA^223^ | 2009 | 5 | 3 | 5 | 1 | 5 | 19 |
| Jago R^82^ | 2005 | 7 | 3 | 5 | 2 | 5 | 22 |
| Janz KF^73^ | 2005 | 7 | 3 | 5 | 1 | 6 | 22 |
| Johnson JG^41^ | 2007 | 6 | 3 | 5 | 1 | 5 | 20 |
| Kaur H^75^ | 2003 | 7 | 3 | 5 | 2 | 5 | 22 |
| Lajunen HR^128^ | 2007 | 8 | 3 | 5 | 2 | 5 | 23 |
| Lonner WJ^238^ | 1985 | 3 | 3 | 5 | 1 | 5 | 17 |
| Maffeis C^89^ | 1998 | 6 | 3 | 5 | 2 | 5 | 21 |
| Mistry K^229^ | 20077 | 9 | 3 | 5 | 2 | 5 | 24 |
| Mitchell JA^49^ | 2009 | 8 | 3 | 5 | 2 | 5 | 23 |
| Must A^87^ | 2007 | 7 | 3 | 5 | 1 | 5 | 21 |
| O'Brien M^69^ | 2007 | 6 | 3 | 5 | 1 | 5 | 20 |
| Parsons TJ^74^ | 2005 | 4 | 3 | 5 | 1 | 5 | 18 |
| Purslow LR^63^ | 2008 | 9 | 3 | 5 | 1 | 5 | 23 |
| Timperio A^65^ | 2008 | 6 | 3 | 5 | 1 | 5 | 20 |
| Treuth MS^29^ | 2007 | 7 | 3 | 5 | 2 | 5 | 22 |
| Wosje KS^205^ | 2009 | 6 | 2 | 5 | 1 | 5 | 19 |
| **Cross sectional** |  |  |  |  |  |  |  |
| Al SH^192^ | 2009 | 6 | 3 | 4 | 1 | 5 | 19 |
| Albarwani S^207^ | 2009 | 5 | 3 | 5 | 1 | 5 | 19 |
| Alves JG^191^ | 2009 | 6 | 1 | 4 | 1 | 5 | 17 |
| Aman J^218^ | 2009 | 6 | 3 | 5 | 1 | 5 | 20 |
| Andersen LF^155^ | 2005 | 8 | 3 | 3 | 2 | 5 | 21 |
| Andersen RE^142^ | 1998 | 5 | 3 | 5 | 1 | 5 | 19 |
| Anderson SE^103^ | 2008 | 7 | 3 | 5 | 1 | 5 | 21 |
| Armstrong CA^213^ | 1998 | 5 | 1 | 5 | 1 | 5 | 17 |
| Asante PA^183^ | 2009 | 7 | 2 | 5 | 2 | 5 | 21 |
| Aucote HM^163^ | 2009 | 5 | 3 | 5 | 1 | 5 | 19 |
| Barlow SE^151^ | 2007 | 7 | 3 | 5 | 1 | 5 | 21 |
| Basaldua N^109^ | 2008 | 7 | 3 | 5 | 2 | 5 | 22 |
| Bellisle^123^ | 2007 | 8 | 3 | 5 | 4 | 5 | 25 |
| Berkey CS^90^ | 2000 | 7 | 3 | 5 | 2 | 5 | 22 |
| Beyerlein A^105^ | 2008 | 6 | 3 | 5 | 1 | 5 | 20 |
| Boone JE^164^ | 2007 | 5 | 3 | 5 | 2 | 5 | 20 |
| Boone-Heinonen J^104^ | 2008 | 5 | 3 | 5 | 1 | 5 | 19 |
| Boutelle KN^130^ | 2009 | 9 | 3 | 5 | 1 | 5 | 23 |
| Brodersen NH^235^ | 2005 | 6 | 3 | 5 | 2 | 5 | 21 |
| Bukara-Radujkovic G^96^ | 2009 | 6 | 3 | 5 | 1 | 5 | 20 |
| Butte NF^119^ | 2007 | 7 | 3 | 5 | 1 | 5 | 21 |
| Caldas SJ^245^ | 1999 | 5 | 3 | 4 | 1 | 5 | 18 |
| Carvalhal^131^ | 2007 | 6 | 3 | 5 | 2 | 5 | 21 |
| Chaput J^154^ | 2006 | 6 | 3 | 5 | 1 | 5 | 20 |
| Chen MY^78^ | 2007 | 6 | 3 | 5 | 2 | 5 | 21 |
| Chowhan J^232^ | 2007 | 8 | 3 | 5 | 1 | 5 | 22 |
| Christoforidis A^95^ | 2009 | 7 | 3 | 5 | 1 | 5 | 21 |
| Collins AE^149^ | 2008 | 6 | 3 | 5 | 1 | 5 | 20 |
| Colwell J^200^ | 2003 | 5 | 3 | 5 | 1 | 5 | 19 |
| Cooper H^247^ | 1999 | 6 | 2 | 5 | 1 | 5 | 19 |
| Crespo CJ^177^ | 2001 | 7 | 3 | 5 | 2 | 5 | 22 |
| Da CR^157^ | 2003 | 7 | 3 | 5 | 2 | 5 | 22 |
| da Silva RC^182^ | 2000 | 4 | 2 | 5 | 1 | 5 | 17 |
| Dasgupta K^215^ | 2007 | 3 | 2 | 5 | 1 | 5 | 16 |
| Delva J^125^ | 2007 | 9 | 3 | 5 | 2 | 5 | 24 |
| Dietz WH^181^ | 1985 | 6 | 3 | 5 | 1 | 5 | 20 |
| Dollman J^211^ | 2006 | 5 | 3 | 5 | 1 | 5 | 19 |
| Dominick JR^225^ | 1984 | 5 | 3 | 5 | 1 | 5 | 19 |
| Dumais SA^255^ | 2009 | 3 | 3 | 2 | 1 | 5 | 14 |
| Eisenmann JC^113^ | 2008 | 8 | 1 | 5 | 1 | 5 | 20 |
| Eisenmann JC^175^ | 2002 | 5 | 3 | 5 | 1 | 5 | 19 |
| Ekelund U^134^ | 2006 | 7 | 2 | 5 | 1 | 5 | 20 |
| Fetler M^249^ | 1984 | 4 | 3 | 5 | 1 | 5 | 18 |
| Forshee RA^188^ | 2009 | 6 | 3 | 5 | 2 | 5 | 21 |
| Forshee RA^201^ | 2004 | 8 | 3 | 5 | 2 | 5 | 23 |
| Gaddy GD^257^ | 1986 | 4 | 3 | 5 | 1 | 5 | 18 |
| Giammattei J^140^ | 2003 | 8 | 3 | 5 | 2 | 5 | 23 |
| Gibson S^156^ | 2004 | 7 | 3 | 5 | 2 | 5 | 22 |
| Gomez LF^150^ | 2007 | 6 | 3 | 5 | 1 | 5 | 20 |
| Gordon-Larsen P^176^ | 2002 | 6 | 3 | 5 | 1 | 5 | 20 |
| Gortmaker SL^147^ | 1996 | 5 | 3 | 5 | 1 | 5 | 19 |
| Gortmaker SL ^57^ | 1999 | 5 | 3 | 5 | 2 | 5 | 20 |
| Graf C^167^ | 2004 | 6 | 3 | 5 | 2 | 5 | 21 |
| Grusser,S.M^40^ | 2005 | 6 | 3 | 5 | 2 | 5 | 21 |
| Hardy LL^133^ | 2006 | 7 | 3 | 4 | 1 | 5 | 20 |
| Hernandez B^178^ | 1999 | 7 | 3 | 5 | 2 | 5 | 22 |
| Hirschler V^144^ | 2009 | 4 | 3 | 5 | 1 | 5 | 18 |
| Holder MD^222^ | 2009 | 5 | 2 | 5 | 1 | 5 | 18 |
| Hume C^190^ | 2009 | 6 | 3 | 4 | 1 | 5 | 19 |
| Islam-Zwart K^195^ | 2008 | 9 | 3 | 5 | 3 | 5 | 25 |
| Jackson LA^223^ | 2009 | 7 | 3 | 5 | 2 | 5 | 22 |
| Janssen I^166^ | 2004 | 7 | 3 | 5 | 2 | 5 | 22 |
| Janz K^174^ | 2002 | 6 | 3 | 5 | 2 | 5 | 21 |
| Jaruratanasirikul S^241^ | 2007 | 7 | 3 | 5 | 2 | 5 | 22 |
| Johnson CC^41^ | 2007 | 9 | 3 | 5 | 1 | 5 | 23 |
| Katzmarzyk PT^197^ | 1998 | 7 | 3 | 5 | 1 | 5 | 21 |
| Katzmarzyk PT^184^ | 1998 | 4 | 3 | 5 | 1 | 5 | 18 |
| Kautiainen S^135^ | 2005 | 6 | 3 | 5 | 1 | 5 | 20 |
| Keith TZ^256^ | 1986 | 3 | 2 | 5 | 1 | 5 | 16 |
| Klein-Platat C^165^ | 2005 | 7 | 3 | 5 | 2 | 5 | 22 |
| Kosti RI^196^ | 2007 | 6 | 3 | 5 | 1 | 5 | 20 |
| Kristjansson AL^243^ | 2009 | 6 | 3 | 5 | 1 | 5 | 20 |
| Kuntsche E^230^ | 2006 | 9 | 3 | 5 | 2 | 5 | 24 |
| Kuriyan R^117^ | 2007 | 6 | 3 | 5 | 2 | 5 | 21 |
| Lagiou A^160^ | 2008 | 5 | 3 | 5 | 2 | 5 | 20 |
| Lajous M^92^ | 2009 | 7 | 3 | 5 | 2 | 5 | 22 |
| Lajunen HR^128^ | 2007 | 8 | 3 | 5 | 2 | 5 | 23 |
| Lasserre AM^116^ | 2007 | 4 | 1 | 5 | 1 | 5 | 16 |
| Laurson KR^107^ | 2008 | 7 | 3 | 5 | 2 | 5 | 22 |
| Lazarou C^217^ | 2009 | 7 | 3 | 5 | 2 | 5 | 22 |
| Leatherdale ST^11^ | 2008 | 9 | 3 | 5 | 1 | 5 | 23 |
| Lioret S^127^ | 2007 | 4 | 3 | 4 | 1 | 5 | 17 |
| Lobelo F^208^ | 2009 | 9 | 3 | 5 | 1 | 5 | 23 |
| Lowry R^173^ | 2002 | 9 | 3 | 5 | 4 | 5 | 26 |
| Lutfiyya MN^118^ | 2007 | 7 | 3 | 5 | 1 | 5 | 21 |
| Maffeis C^114^ | 2008 | 6 | 1 | 5 | 3 | 5 | 20 |
| Mark AE^220^ | 2008 | 8 | 1 | 5 | 2 | 5 | 21 |
| McMurray RG^187^ | 2000 | 9 | 3 | 3 | 0 | 5 | 20 |
| Mihas C^193^ | 2009 | 8 | 3 | 5 | 1 | 5 | 22 |
| Mikolajczyk RT^194^ | 2008 | 6 | 3 | 5 | 2 | 5 | 21 |
| Moraes SA^135^ | 2006 | 4 | 3 | 5 | 1 | 5 | 18 |
| Morgenstern M^94^ | 2009 | 8 | 2 | 5 | 2 | 5 | 22 |
| Mota J^199^ | 2006 | 6 | 3 | 5 | 2 | 5 | 21 |
| Muller MJ^179^ | 1999 | 6 | 3 | 5 | 1 | 5 | 20 |
| Nagel G^193^ | 2009 | 6 | 3 | 5 | 2 | 5 | 21 |
| nastassea-Vlachou K^240^ | 1996 | 8 | 1 | 5 | 1 | 5 | 20 |
| Nawal LM^148^ | 1998 | 4 | 3 | 5 | 1 | 5 | 18 |
| Nelson MC^233^ | 2006 | 7 | 3 | 6 | 4 | 5 | 25 |
| Neumark-Sztainer D^224^ | 2004 | 7 | 3 | 5 | 1 | 5 | 21 |
| Nogueira JA^45^ | 2009 | 6 | 2 | 5 | 2 | 5 | 20 |
| Obarzanek E^180^ | 1994 | 5 | 3 | 5 | 1 | 5 | 19 |
| Ohannessian CM^226^ | 2009 | 5 | 3 | 5 | 2 | 5 | 20 |
| Ortega FB^122^ | 2007 | 9 | 3 | 5 | 1 | 5 | 23 |
| Overby NC^219^ | 2009 | 7 | 3 | 5 | 1 | 5 | 21 |
| Ozmert E^42^ | 2002 | 5 | 3 | 5 | 2 | 5 | 20 |
| Padez C^99^ | 2009 | 6 | 2 | 5 | 1 | 5 | 19 |
| Page RM^234^ | 2001 | 6 | 3 | 5 | 1 | 5 | 20 |
| Pate RR^‑210^ | 2006 | 7 | 3 | 5 | 1 | 5 | 21 |
| Patrick K^169^ | 2004 | 7 | 3 | 4 | 1 | 5 | 20 |
| Pratt C^101^ | 2008 | 7 | 3 | 4 | 1 | 5 | 20 |
| Purath J^185^ | 1995 | 6 | 3 | 5 | 1 | 5 | 20 |
| Ramos E^126^ | 2007 | 6 | 3 | 5 | 1 | 5 | 20 |
| Rapp K^138^ | 2005 | 7 | 3 | 5 | 2 | 5 | 22 |
| Ridley-Johnson R^252^ | 1983 | 5 | 2 | 5 | 1 | 5 | 18 |
| Roberts DF^250^ | 1984 | 3 | 3 | 4 | 1 | 5 | 16 |
| Robinson TN^58^ | 1999 | 7 | 3 | 5 | 1 | 5 | 21 |
| Ruangdaraganon N^141^ | 2002 | 4 | 3 | 4 | 1 | 5 | 17 |
| Russ SA^147^ | 2009 | 8 | 3 | 5 | 1 | 5 | 22 |
| Sakamoto A^236^ | 1994 | 5 | 3 | 5 | 1 | 5 | 19 |
| Salmon,J^136^ | 2006 | 9 | 1 | 3 | 0 | 5 | 18 |
| Sardinha LB^48^ | 2008 | 9 | 3 | 5 | 3 | 5 | 25 |
| Scott LF^254^ | 1958 | 2 | 3 | 5 | 1 | 5 | 16 |
| Sharif I^260^ | 2010 | 5 | 3 | 5 | 1 | 5 | 19 |
| Sharif I^244^ | 2006 | 6 | 3 | 5 | 1 | 5 | 20 |
| Shejwal B^246^ | 2006 | 5 | 3 | 5 | 1 | 5 | 19 |
| Shields M^162^ | 2006 | 8 | 3 | 5 | 2 | 5 | 23 |
| Shin N^239^ | 2004 | 7 | 3 | 5 | 2 | 5 | 22 |
| Singh GK^106^ | 2003 | 6 | 3 | 5 | 1 | 5 | 20 |
| Skoric MM^258^ | 2009 | 7 | 3 | 5 | 2 | 5 | 22 |
| Smith BJ^161^ | 2007 | 7 | 3 | 5 | 1 | 5 | 21 |
| Spinks AB^124^ | 2007 | 8 | 3 | 5 | 2 | 5 | 23 |
| Steffen LM^98^ | 2009 | 7 | 3 | 5 | 2 | 5 | 22 |
| Stettler N^168^ | 2004 | 7 | 3 | 5 | 1 | 5 | 21 |
| Sugiyama T^47^ | 2007 | 7 | 3 | 5 | 1 | 5 | 21 |
| Sun Y^91^ | 2009 | 7 | 3 | 5 | 2 | 5 | 22 |
| Taylor WC^158^ | 2002 | 6 | 1 | 5 | 3 | 5 | 20 |
| te Velde SJ^129^ | 2007 | 7 | 3 | 5 | 1 | 5 | 21 |
| Thompson AM^189^ | 2009 | 7 | 3 | 5 | 1 | 5 | 21 |
| Toschke AM^112^ | 2008 | 9 | 3 | 5 | 2 | 5 | 24 |
| Toschke AM^121^ | 2007 | 4 | 3 | 5 | 1 | 5 | 18 |
| Trang NHHD^146^ | 2009 | 9 | 3 | 3 | 0 | 5 | 20 |
| Tremblay MS^172^ | 2003 | 8 | 3 | 5 | 2 | 5 | 23 |
| Treuth MS^27^ | 2009 | 7 | 3 | 5 | 1 | 5 | 21 |
| Tsai H^145^ | 2009 | 7 | 3 | 5 | 1 | 5 | 21 |
| Tsai H^153^ | 2007 | 6 | 3 | 5 | 2 | 5 | 21 |
| Tucker LA^214^ | 1996 | 6 | 3 | 5 | 1 | 5 | 20 |
| Tucker LA^212^ | 1987 | 6 | 3 | 5 | 1 | 5 | 20 |
| Tucker LA^206^ | 1986 | 5 | 3 | 5 | 1 | 5 | 19 |
| Ussher MH^231^ | 1007 | 8 | 3 | 5 | 1 | 5 | 22 |
| Utter J^152^ | 2007 | 7 | 3 | 5 | 1 | 5 | 21 |
| Utter J^171^ | 2003 | 7 | 3 | 5 | 2 | 5 | 22 |
| Vader AM^97^ | 2009 | 5 | 3 | 5 | 2 | 5 | 20 |
| van Schie EG^261^ | 1997 | 5 | 3 | 5 | 1 | 5 | 19 |
| van Zutphen M^159^ | 2007 | 5 | 1 | 4 | 1 | 5 | 16 |
| Vandewater EA^170^ | 2004 | 8 | 3 | 5 | 1 | 5 | 22 |
| Vaughan C^198^ | 2007 | 5 | 3 | 5 | 1 | 5 | 19 |
| Vicente-Rodriguez G^110^ | 2008 | 7 | 3 | 5 | 2 | 5 | 22 |
| Violante R^137^ | 2005 | 8 | 3 | 5 | 2 | 5 | 23 |
| Wake M^186^ | 2003 | 8 | 3 | 5 | 2 | 5 | 23 |
| Walberg HJ^251^ | 1984 | 6 | 3 | 5 | 1 | 5 | 20 |
| Walberg HJ^253^ | 1982 | 5 | 3 | 5 | 1 | 5 | 19 |
| Waller CE^202^ | 2003 | 9 | 3 | 5 | 2 | 5 | 24 |
| Wang Y^120^ | 2007 | 6 | 3 | 5 | 1 | 5 | 20 |
| Welch WW^248^ | 1986 | 6 | 3 | 5 | 1 | 5 | 20 |
| Wells JC^108^ | 2008 | 6 | 3 | 5 | 2 | 5 | 21 |
| Whitt-Glover MC^24^ | 2009 | 6 | 3 | 4 | 1 | 5 | 19 |
| Wiggins JD^227^ | 1987 | 4 | 3 | 5 | 1 | 5 | 18 |
| Wolf AM^203^ | 1998 | 5 | 3 | 5 | 3 | 5 | 21 |
| Wong SL^100^ | 2009 | 8 | 3 | 5 | 1 | 5 | 22 |
| Zabinski MF^132^ | 2007 | 6 | 3 | 5 | 2 | 5 | 21 |
